# Artificial Intelligence for Forecasting the Prevalence of COVID-19 Pandemic: An Overview

**DOI:** 10.3390/healthcare9121614

**Published:** 2021-11-23

**Authors:** Ammar H. Elsheikh, Amal I. Saba, Hitesh Panchal, Sengottaiyan Shanmugan, Naser A. Alsaleh, Mahmoud Ahmadein

**Affiliations:** 1 Faculty of Engineering, Tanta University, Tanta 31527, Egypt; 2 Faculty of Medicine, Tanta University, Tanta 31527, Egypt; amal.saba@med.tanta.edu.eg; 3 Department of Mechanical Engineering, Government Engineering College, Patan 384265, Gujarat, India; engineerhitesh2000@gmail.com; 4 Research Centre for Solar Energy, Department of Physics, Koneru Lakshmaiah Education Foundation, Vaddeswaram 522502, Andhra Pradesh, India; s.shanmugam1982@gmail.com; 5 Mechanical Engineering Department, Imam Mohammad Ibn Saud Islamic University, Riyadh 11564, Saudi Arabia; naalsaleh@imamu.edu.sa (N.A.A.); maahmadein@imamu.edu.sa (M.A.)

**Keywords:** artificial intelligence, review, COVID-19, forecasting

## Abstract

Since the discovery of COVID-19 at the end of 2019, a significant surge in forecasting publications has been recorded. Both statistical and artificial intelligence (AI) approaches have been reported; however, the AI approaches showed a better accuracy compared with the statistical approaches. This study presents a review on the applications of different AI approaches used in forecasting the spread of this pandemic. The fundamentals of the commonly used AI approaches in this context are briefly explained. Evaluation of the forecasting accuracy using different statistical measures is introduced. This review may assist researchers, experts and policy makers involved in managing the COVID-19 pandemic to develop more accurate forecasting models and enhanced strategies to control the spread of this pandemic. Additionally, this review study is highly significant as it provides more important information of AI applications in forecasting the prevalence of this pandemic.

## 1. Introduction

COVID-19 is a global pandemic that has been rapidly spreading worldwide [1]. It has affected more than 100 million people and caused about two deaths per million as of the end of January 2021. Originating in Wuhan, China, it has spread to about 200 countries. As there is no treatment to this disease, the main way to control this pandemic is by applying strict quarantines and lockdown procedures [2]. Forecasting of the COVID-19 spread has attracted the attention of many scientists, researchers and policy makers, as it helps in developing suitable plans and applying protective measures to combat the spread of the pandemic [3].

Longer-term forecasting of COVID-19 prevalence plays a critical role in making pivotal decisions such as the potential to open universities, schools, houses of worship and workplaces. It may also help to define the precautions and restrictions for citizens to reduce disease transmission. These restrictions should be carefully investigated to minimize their impact on different economic outcomes, such as poverty and unemployment [4]. Moreover, forecasting of COVID-19 prevalence may help in establishing vaccine and therapy plans and selecting hot spots that that should receive more attention from policy makers. Nonetheless, numerous forecasting approaches have been reported in the last two years to predict the spread of the COVID-19 pandemic. Mathematical and statistical based approaches have been widely used to predict the spread of COVID-19 in many countries and regions [5,6]. Apart from statistical and computational forecasting models used to model the spread of COVID-19 pandemic, AI models have recently excelled [7,8]. The outperformance of the AI models over other conventional models has been reported in several investigations [9,10,11,12,13]. This motivated us to prepare this literature review to shed light on the application of AI tools to predict the spread of this pandemic. In this paper, the commonly used models will be briefly explained. Then, the evaluation criteria of the forecasted results will be presented. Finally, a comparative study and discussion of different published articles as well as the future prospects will be discussed.

## 2. Artificial Intelligence Models

The commonly used AI models utilized in modeling the COVID-19 pandemic are introduced in this section. These models are nonlinear autoregressive ANN (NARANN), adaptive neuro-fuzzy inference system (ANFIS), hybrid fractal-fuzzy approach (HFFA), Bayesian neural network (BNN), long short-term memory network (LSTM), variational autoencoder (VAE) and singular spectrum analysis (SSA). The fundamental concepts and mathematical modeling of each approach are briefly explained.

### 2.1. Nonlinear Autoregressive ANN

Nonlinear autoregressive ANN (NARANN) is a special of ANN family that mimics the behavior of biological nervous system. It has been widely used in forecasting of one-dimensional time series for different engineering systems such as industrial production [14], fluctuation of groundwater level [15] and solar radiation [16]. Due to its nonlinear nature, it succeeded to model time series data that has transient nature and high variability [17]. The mathematical formulation of NARNN can be expressed as follows:

(1) $$\xi \left(t\right)=\beta \left(x\left(t-1\right),\xi \left(t-2\right),\ldots .,\xi \left(t-e\right)\right)+\delta \left(t\right)$$

where $\xi \left(t\right)\;$ is the response of time series at time t, and e denotes the delay of the network. $\xi \left(t-1\right),\xi \left(t-2\right),\ldots .,\xi \left(t-e\right)$ represent the historical responses. $\beta \left(.\right)$ is a function with unknown value and it is computed during the training stage by obtaining the optimal bias and weights. $\delta \left(t\right)$ is a time compensation function employed to estimate the error with respect to time.

**Figure 1:**
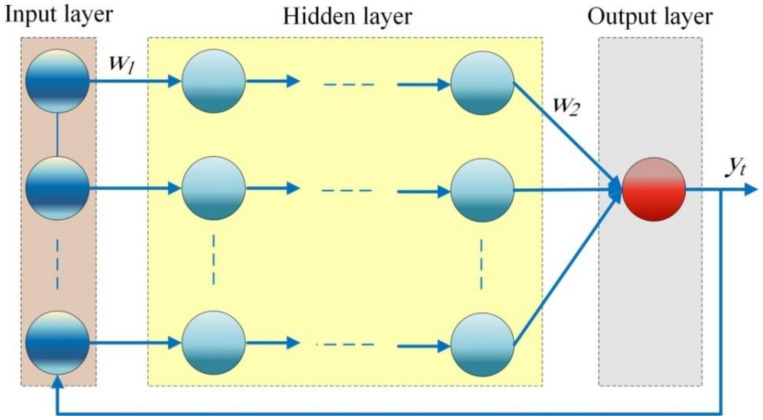
The typical structure of NARANN.

The typical structure of NARANN is presented in Figure 1. It contains an input layer followed by one or multiple hidden layers and an output layer. The number of processing units in the hidden layers and the synaptic weights between them could be adjusted to maximize the forecasting accuracy of the network by obtaining the optimal structure of NARANN. In Figure 1, $\;w_{1}\;\mathrm{and}\;w_{2}\;$are the synaptic weights and $y_{t}$ is the model out output. Increasing the processing units in the hidden layer requires much computational costs and may cause inappropriate over-fitting during a training process, while decreasing the processing units in the hidden layer may result in reducing the model accuracy as well as its generalization capabilities. As with any other ANN, NARANN is trained using real-world data using any training algorithm such as Levenberg-Marquardt backpropagation training algorithm. Once the network is trained, it may be used to predict the behavior of the time series in the future.

### 2.2. Adaptive Neuro-Fuzzy Inference System (ANFIS)

ANFIS is a machine learning tool in which ANN is incorporated with fuzzy logic. It has been used to predict the response of different engineering systems such as drought forecasting [18], tropical cyclones [19] and oil consumption forecasting [20]. It figures out the knowledge between input and output process variables by employing If-Then fuzzy rules.

Each applied rule has antecedent part defined as fuzzy inputs and a consequence part depends on crisp input variables. Fuzzy inputs are combined with each other using logistic mathematical operator (AND operator). The fuzzy rule $F^{r}\;$of a logic system that has n input variables is defined as

(2) $$F^{r}:if\;x_{1\;}is\;B_{1}^{r},x_{2\;}is\;B_{2}^{r}\;,\;\ldots ,x_{i\;}is\;B_{i}^{r},\ldots .and\;x_{n\;}is\;B_{n}^{r}\to$$

$$then\;y^{r}=b_{0}^{r}+b_{1}^{r}x_{1}+\ldots +b_{i}^{r}x_{i}+\ldots +b_{n}^{r}x_{n}\;$$

where $x_{i\;}$ and $y^{r}$ denote the input variable number i and fuzzy output number r, respectively; $B_{i}^{r}$ denotes the membership function, $b_{0}^{r}$ and $b_{i}^{r}$ denote the bias and the regression coefficient, respectively.

**Figure 2:**
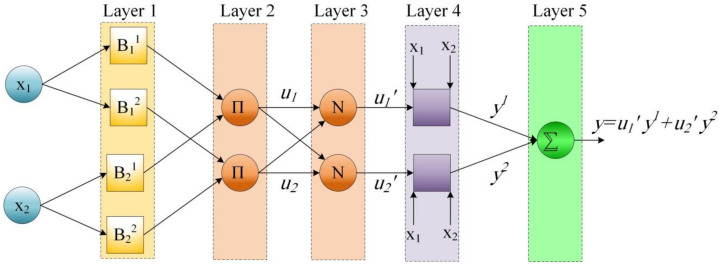
A typical structure of ANFIS.

In a typical ANFIS structure, there are five consequence layers as shown in Figure 2, the tasks of each layer are explained as follows.

In the first layer, membership degrees for each input variable are computed, thus this layer acts as fuzzification layer. Before calculating the membership degrees, the regression coefficients, membership functions and the fuzzy rules must be defined. After defining the primary fuzzy rules, the training process of the ANFIS model is executed. During the training process, the prediction error of the model is minimized via optimizing regression coefficients and membership functions. Two optimization algorithms are utilized to accomplish the optimization process; namely, least-squares algorithm and gradient descend algorithm. The first algorithm is used to optimize the regression coefficients, while the used second algorithm is employed to optimize the internal parameters of the membership functions.

In the subsequent layer, after computing the membership degrees, the antecedent part of fuzzy rules is subjected to the following firing function:

(3) $$u_{r}=\prod_{i=1}^{n}B_{i}^{r}\left(x_{i\;}\right)$$

In the subsequent layer, the normalized weights are computed by

(4) $$u_{r}^{\prime }=\frac{u_{r}}{\sum_{r}u_{r}}$$

In the fourth layer, the consequence value of the applied rules is computed based on the defined regression coefficients as follows:

(5) $$y^{r}=b_{0}^{r}+\sum_{i=1}^{n}b_{i}^{r}x_{i}$$

In the fifth layer, the final output of ANFIS is computed as follows:

(6) $$y=\sum_{r}u_{r}^{\prime }y^{r}$$

### 2.3. Hybrid Fractal-Fuzzy Approach

Hybrid fractal-fuzzy approach (HFFA) us a hybrid approach in which fuzzy logic is integrated with fractal theory. The fuzzy logic based system is used to express the time series knowledge. Fractal theory has shown promising applications in modeling complex engineering problems [21,22]. The main concepts of fuzzy logic and fractal dimension will be discussed in this section.

The fractal dimension of a certain object could be expressed as

(7) $$b=\underset{a\to 0}{\lim}\left[lnE\left(a\right)\right]/\left[ln\left(1/a\right)\right]$$

where $E\left(a\right)$ is the number of boxes employed to shield an object and a denotes a measure parameter of the box size. The fractal dimension is computed as a function of the number of boxes used to shield the object boundary for different a sizes and then b is obtained using logarithmic regression. The box dimensions could be estimated using the following equation:

(8) $$lnE\left(a\right)=ln\beta -blna\;$$

where b denotes the fractal dimension, which could be estimated using a least squares method.

Fuzzy rules could be used as a forecasting tool. First, the input space or time series should be partitioned so that the geometrical objects are distinguished based on their characteristics. Considering the geometrical objects as time series patterns, fuzzy clustering techniques could be applied to cluster the data, then fuzzy rules are constructed. These fuzzy rules will act as a forecasting tool for a certain time series application.

For m objects $OB_{1},OB_{2},\ldots OB_{m}$, clustering using fuzzy technique can be used to obtain pairs $\left(u_{i},v_{i}\right)$ for all m. Then a fuzzy rule is defined by

$$If\;U\;is\;u_{1}\;and\;V\;is\;v_{1}\;then\;Object\;is\;OB_{1}\;If\;U\;is\;u_{2}\;and\;V\;is\;v_{2}\;then\;Object\;is\;OB_{2}\;\ldots \;\ldots \;\ldots \;\ldots \;\ldots \;\ldots \;\ldots \;\ldots \;\ldots \;$$

(9) $$If\;U\;is\;u_{m}\;and\;V\;is\;v_{m}\;then\;Object\;is\;OB_{m}$$

These fuzzy rules can be used in time series prediction.

In case of executing time series forecasting, data analysis should be carried out to explore the periodicities and trends within the data. Then, the time series is clustered into m objects $OB_{1},OB_{2},\ldots OB_{m}$. Objects $OB_{1},OB_{2},\ldots OB_{m}$ is classified based on their geometrical complexity into L or N for linear and non-linear, respectively. Assume the time series has the form $\;v_{1},v_{2},\ldots v_{m}.$ Then, the fuzzy rules are expressed in a general form for time series forecasting as follows:

$$If\;L\;is\;u_{1}\;and\;N\;is\;v_{1}\;then\;Object\;is\;OB_{1}\;\;If\;L\;is\;u_{2}\;and\;N\;is\;v_{2}\;then\;Object\;is\;OB_{2}\;\ldots \;\ldots \;\ldots \;\ldots \;\ldots \;$$

(10) $$If\;L\;is\;u_{m}and\;N\;is\;v_{m}\;then\;Object\;is\;OB_{m}$$

Consequently, the membership functions are defined for the two different fractal dimensions, namely, linear and non-linear.

### 2.4. Long Short-Term Memory Network (LSTM)

LSTM is a well-known recurrent neural network (RNN). Like any other RNN, LSTM has many identical modules connected with each other to form a chain architecture, which is utilized as an artificial memory to process and store data [23]. LSTM is widely used in forecasting time series because of its deep learning capabilities that enable it to memorize and learn sequential evidence. It has superior advantages over other RNN models as it overcomes the main problems of RNN, such as vanishing/exploding gradient and instability. Moreover, LSTM has a good ability to detect patterns in time series, such as trends, autocorrelations, seasonality, and noise. It succeeded to predict different engineering time series such as explosibility of underground mines [24], electricity load [25], soil behavior [26], water yield of solar stills [27] and air pollution [28].

**Figure 3:**
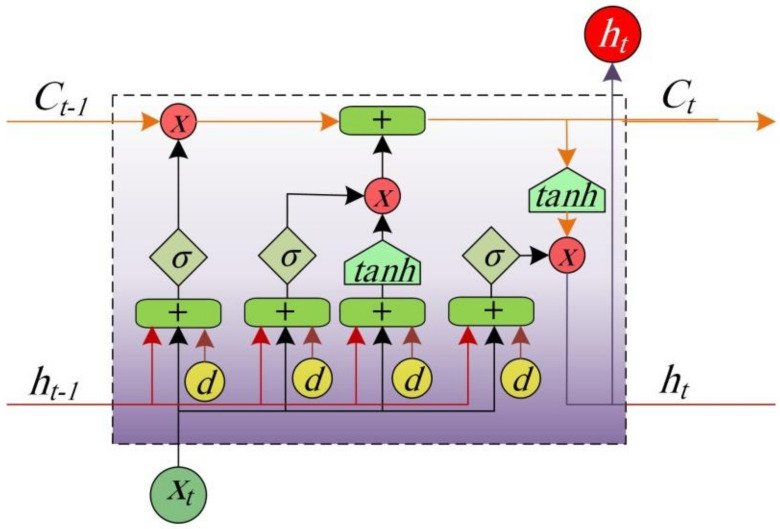
A typical LSTM cell.

LSTMs can learn dependencies found in data and can remember huge amounts of information. A typical LSTM model consists of multiple modules connected in a chain manner. LSTM network has a combined short-term and long-term memory and has the ability to preserve cell state. The key parameter of the LSTM network is the memory cell. A typical LSTM cell, which acts as a fundamental network unit is shown Figure 3. The existence of input, output and forgot gates enhances the learning capabilities of LSTM.

LSTM network identifies the trivial evidence that may be omitted from the processed data. The importance of certain information within the data is identified via passing the data through a sigmoid activation function, which acts as a forget gate as it removes the trivial part of the processed data. This function has an output $f_{t}$ defined as follows:

(11) $$f_{t}=\sigma \left(U_{f}\left[h_{t-1},X_{t}\right]+d_{f}\right)\;$$

where σ is the sigmoid function, $d_{f}$ is the applied bias at the gate and $U_{f}$ is the weight matrix of the gate.

The input $Y_{t}\;$of the following module is passed through two activation functions: tanh and sigmoid. These functions make a decision on saving or omitting information from the data. The sigmoid function makes a decision regarding excluding or updating the new input (0 or 1). The tanh function defines the updated weight of the input and assigns it a significance index with numerical values between −1 and 1. The computed output of the two functions produces the new cell state $C_{t}$ by adding it to the old memory to $C_{t-1}$. These calculations are performed as follows:

(12) $$S_{t}=\sigma \left(U_{i}\left[h_{t-1},X_{t}\right]+d_{i}\right)\;$$

(13) $$T_{t}=tanh\left(U_{n}\left[h_{t-1},X_{t}\right]+d_{n}\right)\;$$

(14) $$C_{t}=C_{t-1}f_{t}+T_{t}S_{t}$$

where, $C_{t-1}$ and $C_{t}$ denote the cell states at time t−1 and t, respectively, $d_{n}$ denotes the applied bias at the gate, and $U_{n}$ is the weight matrix of the gate.

The final output $O_{t}\;$is given by;

(15) $$O_{t}=\sigma \left(U_{o}\left[h_{t-1},X_{t}\right]+d_{o}\right)\;$$

where, $d_{o}\;$is the applied bias at the gate, and $U_{o}$ is the weight matrix of the gate.

### 2.5. Bayesian Neural Network (BNN)

BNN is an advanced ANN integrated with an inference system, which is used in many applications such as electricity load forecasting [29], wind power forecasting [30] and hydrologic forecasting [31]. It overcomes the problems related to conventional ANN, such as over-fitting caused by the uncertainty of synaptic weights estimation. The Bayesian-based training algorithm may help in solving these problems. It applies a probability density function on the network weight space. This function is used to express the belief degree of the weight vector. It is initialized by equating it with a prior distribution. Then, the data is processed via the application of Bayes’ theorem. Finally, the function is converted into a new posterior distribution. Therefore, instead of computing the best weights by conventional approaches via executing a minimization process of a defined error function, the Bayesian approach estimates a whole distribution for the network parameters. The determined posterior distribution is utilized as an inference prediction tool to forecast the new responses of the new input variables.

**Figure 4:**
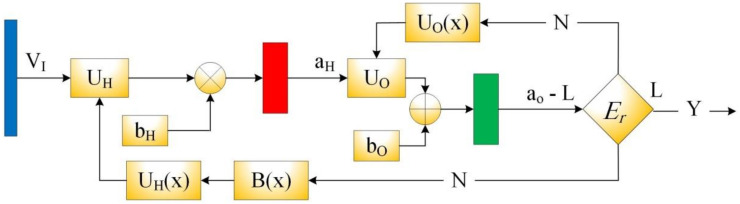
The training of BNN.

The training process of BNN is presented in Figure 4. The key target of the training process is to reduce the error between the predicted values and the real world values. This minimization process is defined by applying some rules: $B\left(x\right)$, $U_{O}\left(x\right)$ and $U_{H}\left(x\right)$ until the stopping criteria $E_{r}\;$is fulfilled. This process could be represented mathematically un the following form:

(16) $$L\left(V_{I}\right)=L\left(V_{I}\right)+\min\left(f_{O}\left(f_{H}\left(V_{I}.U_{H}+b_{H}\right).U_{O}+b_{O}\right)-L\left(V_{I}\right)\right)\;$$

where $L\left(V_{I}\right)$, $U_{H}$,$\;U_{O}$,$\;b_{H}$ and $b_{O}$ denote the real value, the matrix of hidden layer weights, the matrix of output layer weights, the vector of bias of the hidden layer and the vector of bias of the output layer, respectively. $f_{H}\left(.\right)$ and $f_{O}\left(.\right)$ are the hidden and output functions, respectively, which are given by

(17) $$f_{H}\left(x\right)=\frac{2}{1+\exp\left(-2x\right)}-1$$

(18) $$f_{O}\left(x\right)=x\;$$

Once the forecasting model is established, it may be used to forecast the future behavior of time series using the following equation:

(19) $$L\left(IV\right)=f_{O}\left(f_{H}\left(IV.U_{H}+b_{H}\right).U_{O}+b_{O}\right)\;$$

where IV denotes the latest inputs values in a vector form and $L\left(IV\right)$ denotes the forecasted vector.

### 2.6. Variational AutoEncoder (VAE)

**Figure 5:**
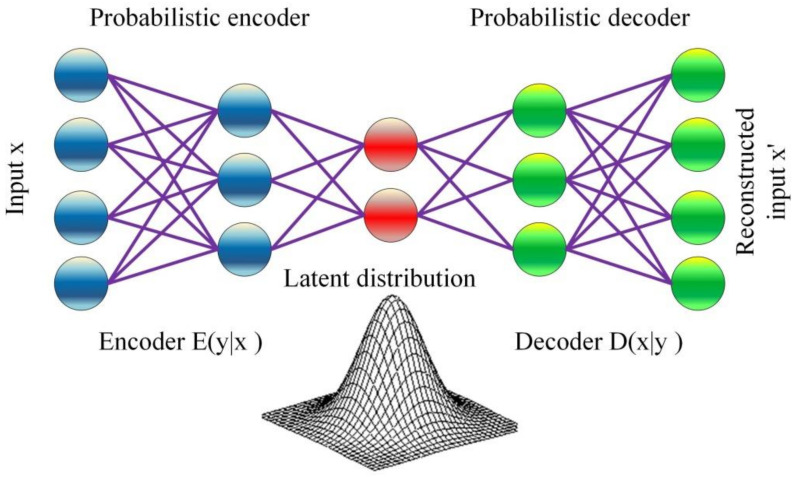
The architecture of VAE.

VAE is a machine learning technique that exploits the advantage of learned inference and could be established using gradient-based methods. The term variational represents the variational inference technique, which belongs to statistics-based methods. The term auto-encoder represents the unsupervised ANN model used to learn data coding. VAE is an auto-encoder model with enhanced training algorithm, which overcomes over-fitting problem by using a special regularization technique. It also creates a latent space with appropriate characteristics that permit the executive of efficient generative process. The basic architecture VAE is shown in Figure 5. VAE has a decoder (shown in green in the figure) and an encoder (shown in blue in the figure) like any other conventional auto-encoder. During the training of the model, the reconstruction error between the input data and the reconstructed encoded–decoded data is minimized. The input data is passed through the encoder E(y|x) via the latent space. At that time, y ~ E(y|x), is created from the code distribution. After that, the coded point is decoded by passing through the decoder D(x|y). Finally, the reconstruction error is computed and the network is updated via a back-propagation process.

The training process is accomplished via minimizing a loss function $f\left(e\right)$ that consists of a reconstruction term and a regularization term. The first term is used to enhance the encoding–decoding process, while the second term is used to regularize the latent space structure. The latent distribution is chosen to match a standard distribution, such as Gaussian distribution. The loss function is given by:

(20) $$f\left(e\right)=G_{y\sim E\left(yx\right)}\left(\log\left(xy\right)\right)-KL\left(E\left(y|x\right)||D\left(y\right)\right)$$

The reconstruction term $G_{y\sim E\left(yx\right)}\left(\log\left(xy\right)\right)$ enables the decoder to learn the reconstruction of the training data. The regularization term is expressed as a function of the Kulback–Leibler $KL\left(.\right)$ divergence between the distribution of the encoder $E\left(y|x\right)$ the latent sample $y,\;|D\left(y\right)$. The $KL\left(.\right)$ measures the loss when E is used to represent D.

### 2.7. Singular Spectrum Analysis (SSA)

SSA is an advanced time series analysis tool, which is classified as a nonparametric spectral estimation technique [32]. It has been used in forecasting different engineering time series such as annual precipitation and hourly water temperature [33], mutual investment funds [34], wind speed [35] and electricity consumption [36]. In a typical SSA model, a conventional time series analysis is integrated with signal processing, multivariate geometry, multivariate statistics and dynamical systems. It has two main phases. The first is the decomposition phase in which the time series is broken down into several components, such as cyclical, seasonal and trend components. This phase facilities the extraction of signals and a reduction of related noise. The second is the reconstruction phase in which data forecasting is accomplished via reconstructing the decomposed components.

The decomposition phase is accomplished via executing two steps: embedding and singular value decomposition. During the embedding step, the time series $\left\{y_{1}\;\;y_{2}\ldots y_{M}\right\}\;$is converted into a trajectory matrix Y with dimension N×L. Each column in this matrix has the form of $Y_{1}=\left(y_{i\;}\;y_{i+1\;}\ldots \;y_{i+N-1\;}\right)$, where L=M−N+1. During the decomposition, the established trajectory matrix Y is decomposed into $Y=\Psi \Omega \Phi^{T}$ where Ψ and Φ are two orthogonal matrices and Ω is a diagonal matrix. The diagonal elements of Ω are $\tau_{1}\;\tau_{2}\ldots \tau_{N}$, which represent the singular values of Y. The decomposed trajectory matrix is given by

(21) $$Y=Y_{1}+Y_{2}+\ldots +Y_{d}$$

where d is the maximum i such that $\tau_{i}\geq 0$ and $Y_{i}=\tau_{i}+\Psi_{i}\Phi_{i}^{T}$.

The reconstruction phase is accomplished via executing two stages: grouping and diagonal averaging. In the grouping stage, the elementary matrices$\;Y_{i}\;$is divided into many groups and the generated matrices within each new group are summed. For a certain group I, a new matrix$\;Y_{I}\;$is formed and defined as $\;Y_{I}=Y_{i1}+Y_{i2}+\ldots +Y_{ip}$. By applying singular value decomposition Y, the following decomposition is obtained:

(22) $$Y=Y_{I1}+Y_{I2}+\ldots +Y_{IM}$$

In the diagonal average stage, each decomposed matric $Y_{I_{J}}$ is transformed into a Hankel matrix. The transformed matrix can be transformed into a time series form with a length M. To obtain the following expansion $Y={\tilde{Y}}_{I_{1}}+{\tilde{Y}}_{I_{2}}+\ldots .+{\tilde{Y}}_{I_{M}}$ with ${\tilde{Y}}_{I_{i}}=HY_{I_{i}}$ with new elements ${\tilde{y}}_{s}\;$over the anti-diagonals computed by ${\tilde{y}}_{s}=\underset{\left(l,n\right)\in Y_{s}}{\sum }\frac{y_{ln}}{\left|Y_{c}\right|}$, where $Y_{s}=\left\{\left(l,n\right):l+n=c+1,1\leq l\leq L,1\leq n\leq N\right\}$ and $\left|Y_{c}\right|$ is the number of elements in $Y_{c}$.

The forecasting process could be accomplished with two main processes, namely, recurrent forecasting and vector forecasting.

The recurrent forecasting is executed according to the following procedures:

1. The new time series $X_{M+h=}\left\{x_{1},x_{2},\ldots .,x_{M+h}\;\right\}$ is defined as follows:
  (23) $$x_{i}=\left\{\begin{array}{c} {\tilde{Y}}_{i},\;for\;i=1,2,\ldots .,\;M\;\\ \sum_{j=1}^{N-1}e_{j}x_{i-j},\;for\;i=M+1,M+2,\ldots .,\;M+h \end{array}\right.$$
  where the coefficients vector $V={\left(e_{N-1\;},e_{N-2,\;}\ldots .,\;e_{1,\;}\right)}^{T}$ is computed as follows:
  (24) $$V=\frac{1}{1-\varphi^{2}}\sum_{i\in I}^{\;}\pi_{i}\Psi_{i}$$
2. The numbers $x_{L+1},\;x_{L+2},\;\ldots ,x_{L+h}\;$ represent the recurrent forecast with an h step.

The vector forecasting is executed according to the following procedures:

1. Define a new time series vector U as follows;
  (25) $$U_{i}=\left\{\begin{array}{c} {\overset{ˇ}{Y}}_{i},\;for\;i=1,2,\ldots .,\;L\;\\ \Upsilon U_{i-1},\;for\;i=L+1,L+2,\ldots .,\;L+h+M-1 \end{array}\right.$$
  where ${\overset{ˇ}{Y}}_{i}=\underset{j\in I}{\sum }\Psi_{j}\Psi_{j}^{T}Y_{i}$,$\;\Upsilon U_{i-1}=\left(\begin{array}{c} \Gamma {\bar{U}}_{i-1}\\ V^{T}{\bar{U}}_{i-1} \end{array}\right)$, $\Gamma =\Phi \Phi^{T}+\left(1-\varphi^{2}\right)\;\Upsilon \Upsilon^{T}$, and ${\bar{U}}_{i-1}\;$represent a vector of $U_{i-1}$ last components.
2. The time series $\left\{x_{1},x_{2},\ldots .,x_{M+h+N-1}\;\right\}$ is obtained by averaging the diagonal of constructed matrix $U=\left[U_{1}:\ldots .:U_{L+h+M-1}\right]$.
3. The numbers $x_{L+1},\;x_{L+2},\;\ldots ,x_{L+h}\;$represent the vector forecast with an h step.

## 3. Evaluation Criteria

The statistical metrics are used as indicators to evaluate the performance of AI models. The most common used statistical metrics are root mean square error (RMSE), normalized root-mean-square error (NRMSE), coefficient of variation (COV), coefficient of determination (R^2^), mean absolute error (MAE), mean absolute percentage error (MAPE), overall index (OI), efficiency coefficient (EC), mean relative error (MRE), and residual mass coefficient (RMC) [37].

As the numerical value of RMSE, NRMSE, MRE, MAE, MAPE, RMC and COV being a small, the discrepancy is small and the model accuracy increases. They can be calculated as

(26) $$RMSE=\sqrt{\frac{1}{n_{s}}\sum_{i=1}^{n_{s}}{\left(d_{i}-y_{i}\right)}^{2}}\;$$

(27) $$NRMSE=\frac{\sqrt{n_{s}\sum_{i=1}^{n_{s}}{\left(d_{i}-y_{i}\right)}^{2}}}{\sum_{i=1}^{n_{s}}y_{i}}\;$$

(28) $$MRE=\frac{1}{n_{s}}\sum_{i=1}^{n_{s}}\frac{d_{i}-y_{i}}{d_{i}}\;$$

(29) $$MAE=\frac{1}{n_{s}}\overset{n_{s}}{\underset{i=1}{\sum }}\left|d_{i}-y_{i}\right|\;$$

(30) $$MAPE=\frac{1}{n_{s}}\overset{n_{s}}{\underset{i=1}{\sum }}\frac{\left|d_{i}-y_{i}\right|}{\left|d_{i}\right|}\;$$

(31) $$RMC=\frac{\sum_{i=1}^{n_{s}}y_{i}-\sum_{i=1}^{n_{s}}d_{i}}{\sum_{i=1}^{n_{s}}d_{i}}$$

(32) $$COV=\left(\left(\frac{RMSE}{\bar{y}}\right)\;\times 100\right)\;$$

As the value of R^2^, OI and EC increases (approaches the unity), the model has high accuracy. They are calculated by

(33) $$R^{2}=\frac{{\left(\sum_{i=1}^{n_{s}}\left(d_{i}-\bar{d}\right)\left(y_{i}-\bar{y}\right)\right)}^{2}}{\sum_{i=1}^{n_{s}}{\left(d_{i}-\bar{d}\right)}^{2}\times \sum_{i=1}^{n_{s}}{\left(y_{i}-\bar{y}\right)}^{2}}\;$$

(34) $$EC=1-\frac{\sum_{i=1}^{n_{s}}{\left(d_{i}-y_{i}\right)}^{2}}{\sum_{i=1}^{n_{s}}{\left(d_{i}-\bar{d}\right)}^{2}}\;$$

(35) $$OI=\frac{1}{2}\left(1-\left(\frac{RMSE}{d_{max}-d_{min}}\right)+EC\right)\;$$

where, *n_s_*, *d*, and *y* denote the number of iterations, actual and the forecasted data, respectively. $d_{min}$, $d_{max}$, and $\bar{d\;}$ represent the minimum, maximum and mean values of the target data, while $\bar{\;y}$ represents the mean value of the predicted dataset.

## 4. Comparative Study and Discussion

In this section, the published studies on the applications of AI techniques for forecasting COVID-19 spread are discussed. The reported comparisons between different AI techniques as well as conventional statistical techniques are also presented.

### 4.1. Statistical Models

Many forecasting studies have been carried out by employing statistical and mathematical approaches to predict the prevalence of COVID-19 pandemic. An auto-regressive integrated moving average (ARIMA) model was employed to predict COVID-19 spread for one month in Pakistan [38]. The increase rate of confirmed cases, recoveries and deaths has an exponential trend. The same approach was applied to predict the COVID-19 cases in South Korea and Japan [39]. A hybrid ARIMA model incorporated with wavelet-based approach was developed to predict the daily cases in India, South Korea, Canada, the United Kingdom and France on a real-time basis [40]. A high correlation between the mortality rate and four control variables, namely, total number of cases, age, lockdown period and total number of hospitals have been reported. Another hybrid model composed of discrete wavelet and ARIMA model was developed to predict the pandemic spread in Spain, Italy, France, the United Kingdom, and United States [41]. A modified version of ARIMA incorporated with α-Sutte indicator and called SutteARIMA has been developed to predict the total cases of COVID-19 in Spain [42] and another modified version incorporated with Kalman filter has been developed to predict the total active cases, deaths and recoveries in Pakistan [43]. A comparison investigation has been carried out between ARIMA model, support vector regression (SVR), ridge regression, random forest, cubist regression and stacking-ensemble learning as statistical-based models to assess their capabilities to predict total confirmed cases in Brazil [44]. Support vector regression and stacking-ensemble learning showed the best forecasting accuracy among the other investigated models. Gaussian spreading hypothesis have been employed to predict the epidemiological behavior in eight countries, namely, Greece, Germany, Netherlands, Spain, Italy, France, the United Kingdom and the United States based on the historical data reported by the Chinese government [45]. It has been reported that applying strict measures has a significant role in reducing the disease spreading. Reduced-space Gaussian regression modeling was applied to predict the spreading of the pandemic in United States via applying a hybrid incorporation between Bayesian system and geographic information system [46]. Another mathematical-based epidemiological model called susceptible/infectious/recovered model was employed to predict the transmission dynamics of the COVID-19 outbreak in China, Italy, Spain, Russia, Germany, France, South Korea and the United States [47]. This model is used to predict the infected cases as a time series, disease spread, pandemic duration, and the disease reproductive number.

The dynamic epidemiological behavior of the COVID-19 outbreak in three Chinese cities, namely, Beijing, Shenzhen and Guangzhou has been investigated using the ecological niche models [48]. Nine socioeconomic factors have been considered, namely, population density, pandemic data of infected communities, number of subway stations, number of bus stops, total length of roads, number of supermarkets, number of shopping malls, number of hospitals, number of appointed hospitals and average prices of rental houses. It was recommended that local hygienic authorities should adjust their interventions in the COVID-19 hotspots with high population density.

Grey prediction models have also been employed to predict the spread of COVID-19 pandemic [49]. A comparison investigation has been carried out between three grey models, namely, grey model, fractional nonlinear grey Bernoulli model, and nonlinear grey Bernoulli model, to evaluate their accuracy to predict the total number of cases in Italy, the United Kingdom and the United States. Fractional nonlinear grey Bernoulli model showed the best prediction accuracy compared to the others.

COVID-19 infected cases was predicted by fitting asymptotic distributions to the reported data [50]. The proposed approach was employed to compare between the pandemic behavior of the outbreak in Italy and China. Death rate due to the pandemic in Italy was predicted using patient information based algorithm [51]. A new iterative method was proposed to predict daily growth rate of confirmed cases in four countries (United States, Germany, Slovenia, and Iran) based on the reported cases, recoveries and deaths [52]. Exponential approach was proposed to predict the total cases outside China; a heuristic computing approach was employed to determine exponential curve parameters.

The total reported cases in Nigeria was estimated using different statistical models, namely, simple linear, quadratic, quartic, cubic, logarithm linear, logarithm quadratic, logarithm quartic, logarithm cubic and inverse linear regression models [53]. The quartic regression model was reported as the best accurate model, among others. The model parameters were calculated using four different approaches, namely, least absolute deviation, Cochrane Orcutt, Hildreth–Lu, ordinary least squares, and Prais-Winsten. The least absolute deviation approach was reported as the best one among others in terms of accuracy, robustness and speed.

Two mathematical models were developed to determine the variations in the daily reported cases [54]. In the first model, the confirmed cases were computed based on the total number of active cases, the number of infected persons people were in close contact with, the rate of performed tests and the mortality rate. In the second model, the Fourier decomposition technique was employed to decompose the COVID-19 time-series in terms of cosine and sine functions. The models were applied to predict the total cases and its peak value in United States, Italy, and India. The SIDARTHE dynamical model was employed to model COVID-19 pandemic [55]. The population of infection is divided into five population subcategories, namely, infected, diagnosed, ailing, recognized and threatened. The effects of the employed control strategies, such as vaccination and different treatment strategies on the infection population subcategories, were investigated.

However, there are many statistical approaches reported in the literature to predict COVID-19 spread. Most of these approaches have been reported to have lower accuracy compared with AI approaches based on different statistical indicators. Moreover, most of approaches are valid only for short-term forecasting. On the other hand, AI approaches succeeded to forecast the COVID-19 spread for both short and long terms.

### 4.2. Artificial Intelligence Models

**Figure 6:**
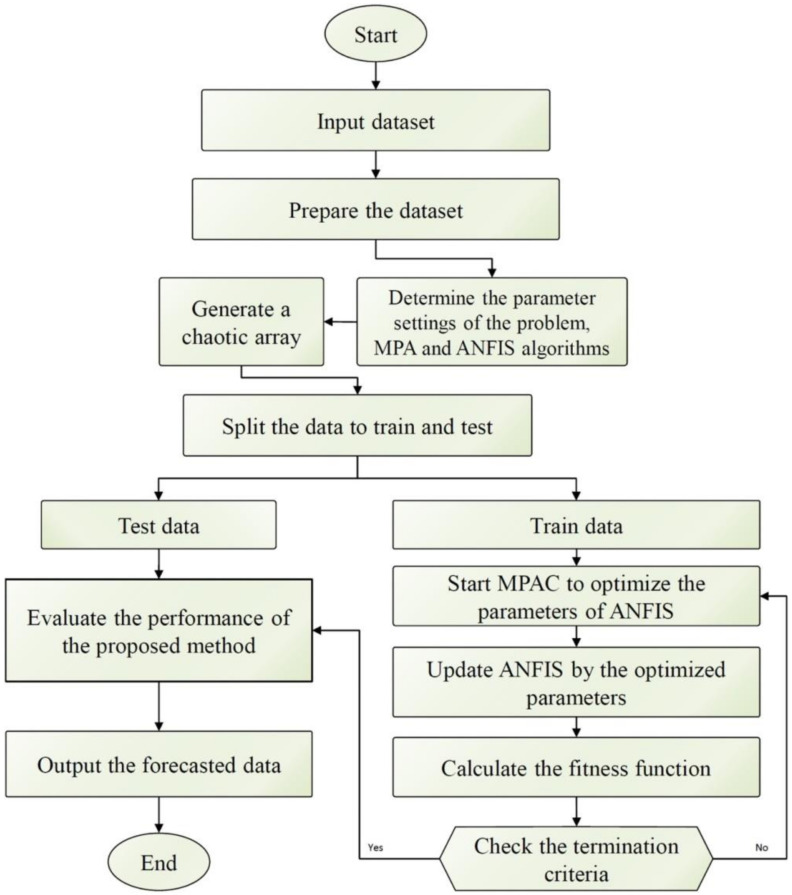
The flow chart of the hybrid ANFIS/chaotic marine predators algorithm used as an optimized forecasting tool [59].

AI models have been rapidly developed to predict COVID-19 spread. The commonly used AI models utilized in modeling the COVID-19 pandemic are introduced in Section 2. These models are NARANN, ANFIS, HFFA, LSTM, BNN, VAE and SSA. ANFIS has been applied to predict the future number of COVID-19 cases in the United Kingdom [56] and Malaysia [57]. In the first study, a parametric comparison has been carried out to obtain the optimal ANFIS model based on model accuracy considering different model parameters. To enhance the forecasting accuracy of ANFIS, it has been integrated with a virus optimization algorithm to forecast COVID-19 cases in the United States [58]. Virus algorithm gas been used to optimize the membership functions and the regression coefficients instead of conventional optimization techniques, which have tendency to be trapped into local optima. The effects of population density and climatology factors on the pandemic spread were investigated. Population density was reported as a major factor that affects the infection rate. In another study, ANFIS has been integrated with chaotic marine predators algorithm to obtain its optimal parameters that maximize the forecasting accuracy [59]. The flow chart of the hybrid ANFIS/chaotic marine predators algorithm is shown in Figure 6. The results of the proposed algorithm have been compared with that of standalone ANFIS as well as the optimized ANFIS using particle swarm optimization. The proposed algorithm had better forecasting accuracy; however, it had the highest computational cost among other investigated algorithms.

HFFA has been employed for forecasting the cases and deaths of COVID-19 considering four main inputs, namely, confirmed cases with linear fractal dimension, confirmed cases with nonlinear fractal dimension, deaths with linear fractal dimension and deaths with nonlinear fractal dimension [60]. The fractal dimension was utilized to determine the complexity of the time series, while fuzzy logic was utilized to characterize the forecasting uncertainty. The proposed approach was used to predict the COVID-19 spread in 10 countries in Europe, Asia and America for a long forecasting period (one month).

SSA was employed to predict the confirmed case in the top 10 affected countries (United States, Brazil, India, France, Russia, Spain, United Kingdom, Mexico, Colombia and Argentina) considering both vector and recurrent forecasting algorithms [9]. The obtained results by SSA were compared by those obtained by ARIMA, ETS, NNAR, ARFIMA and TBATS. The evidence from that study indicated that there is not a single algorithm that may be considered as best for all investigated countries, which have different pandemic trends.

Recurrent SSA has been also used in forecasting COVID--19 cases in Malaysia [61]. The obtained results indicated that the proposed model is able to predict the spread of the outbreak with reasonable accuracy as it has over-forecasted by 0.36% with high correlation between predicted and confirmed cases. However, it cannot capture the sudden variation in the COVID-19 spread caused by the applied motion control strategies.

BNN was employed to predict the accumulated confirmed cases in five American states and five Brazilian states [62]. Moreover, it was reported that including the metrological conditions, such as precipitation and temperature, enhances the accuracy in the forecasting models. BNN had a higher forecasting accuracy compared with other investigated standalone models, such as support vector regression, cubist regression, quantile random forest, and k-nearest neighbors.

A hybrid approach composed of an RVFL model and a discrete wavelet transform was developed to predict the spread of COVID-19 in Russia, Brazil, Peru, India, and the United States [63]. The forecasting accuracy of the proposed approach is compared with the SVR model and the standalone RVFL model. The approach succeeded to obtain accurate forecasting for long period (60 days) and showed better performance compared with other models.

LSTM has been applied to predict COVID-19 cases in Canada [64], India [65], India, the United States [66], and Peru, Russia and Iran [67]. It succeeded to predict the trend of the pandemic despite of the nature of the transmission rate trend (linear, cubic, or exponential growth).

Three LSTM architectures, namely, stacked LSTM, convolutional LSTM and bi-directional LSTM have been investigated and compared with each other to predict the cumulative infected cases in India and the United States for one month ahead [66]. The convolutional LSTM model had the best accuracy followed by bi-directional, while the stacked LSTM model had the worst accuracy.

**Figure 7:**
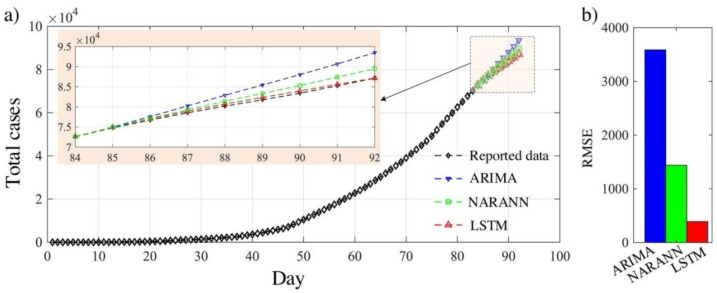
(**a**) Time series plot of the reported COVID-19 cases and forecasted data by NARANN, ARIMA and LSTM; (**b**) the RMSE for three approaches [11].

A comparative study between ARIMA, LSTM and NARNN has been carried out to predict COVID-19 cases in eight European countries, namely, Germany, the United Kingdom, Denmark, France, Belgium, Finland, Turkey and Switzerland [12]. Despite of the differences in human behavior, applied measures and available data for each country, LSTM had much higher forecasting accuracy compared to ARIMA and NARNN for all countries investigated within that study. In another study conducted on Saudi Arabia data, the three approaches were compared [11]. ARIMA and NARANN had a higher forecasting error compared with LSTM as their RMSE are higher than that of LSTM by 909% and 357%, respectively. Thus, LSTM has outperformance over ARIMA and NARNN in forecasting COVID-19 cases, as shown in Figure 7.

Transfer learning has been applied in LSTM networks to predict the spread trend of COVID-19 cases and deaths in France, Germany, India, Brazil and Nepal based on the data of early infected countries such as the United States and Italy [68]. The network was trained using data of early infected countries. LSTM succeeded to capture the complex patterns of the pandemic spread in the United States and Italy and in utilizing them in the forecasting process. LSTM has been also used to predict the number of Chinese tourists that travel to the United States and Australia during the COVID-19 pandemic based on the tourism data collected after the 2003 SARS outbreak [69]. It was reported that the delay of vaccine discovery would significantly decelerate the economic recovery. In addition, the application of preventive measures could affect the prediction accuracy of the proposed approach.

**Figure 8:**
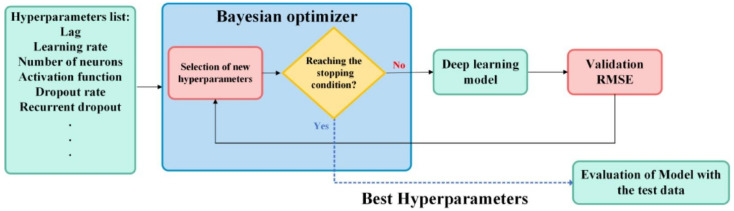
The hybrid LSTM/Bayesian optimization technique for forecasting COVID-19 data [70].

An optimized version of LSTM has been proposed by [70] to predict the spread of the COVID-19 pandemic. The Bayesian optimization technique was used to select the optimal LSTM parameters, such as activation function, learning rate and number of neurons. The hybrid LSTM/Bayesian optimization technique is shown in Figure 8. The proposed method has been used for short and long horizon forecasting. The optimized LSTM outperforms the standalone LSTM as well as the optimized version of CNN.

The number of daily positive cases, recovered cases and deceased cases in India was forecasted using LSTM and curve fitting approach [71]. The effect of the applied preventing measures, such as hotspots lockdown and social distancing on the outbreak spreading, have been investigated. The spread of the outbreak can be significantly reduced by applying strict preventive measures.

A comparison study between ANN and two mathematical approaches, namely, logistic and Gompertz, to predict the total cases in Mexico was carried out by [72]. A good fit between the reported data and those predicted by ANN, logistic, and Gompertz models with correlation coefficient of 0.9999, 0.9996, and 0.9998, respectively. It was declared that the forecasted results may significantly differ from the reported ones, if social distancing policies and testing strategies would be changing in the coming days. Recurrent ANN model was developed to predict the daily mutation rate of COVID-19 [73]. The developed model was applied to the United States, Australia, China and the world. A comparison investigation was carried out to evaluate the forecasting accuracy of three commonly used models: ARIMA, nonlinear autoregression neural network and LSTM ANN [12]. The results of the three investigated models were compared with actual reported data and the forecasting accuracy was evaluated using different statistical criteria. LSTM ANN showed the best accuracy among all investigated models to predict the total cases in eight countries, namely, Switzerland, Finland, Germany, Denmark, France, Turkey, Belgium, and the United Kingdom. In another study, LSTM ANN has been employed to predict the infected cases in Canada [64]. The spread of the pandemic in Canada is lower than that of the United States and Italy, which reveals the effective control policies employed by the Canadian government. The transmission of the disease in Canada has a linear trend in contrary to that of the Unites States and Italy, which has an exponential trend. Based on the obtained results, the COVID-19 pandemic was expected to end by December 2020. The number of confirmed total cases in Egypt was forecasted using nonlinear autoregressive artificial neural networks, which showed a better forecasting accuracy compared with conventional autoregressive integrated moving average approach [10].

A novel hybrid approach consists of LSTM optimized by grey wolf optimizer for forecasting the future cumulated infected cases in the United States, India and the United Kingdom was proposed by [74]. The proposed approach utilized Google Trends and official reported data of the pandemic spread in training the network. The hybrid approach outperformed other conventional statistical approaches, such as ARIMA.

Five artificial intelligence approaches, namely, RNN, LSTM, bi-directional LSTM, GRUs and VAE have been developed to forecast recovered and confirmed cases in six different countries (United States, China, Italy, France, Spain, and Australia) based on short-period collected data [75]. VAE had better forecasting accuracy compared with other proposed approaches for all countries.

Metaheuristic optimization approaches have been also used to predict the prevalence of this pandemic [76]. A genetic-based programming approach has been developed to predict the number of total cases and total deaths in three Indian states, namely, Gujarat, Maharashtra and Delhi [77]. An enhanced ANFIS incorporated with two metaheuristic optimization algorithms, flower pollination and salp swarm algorithms, has been developed to predict the prevalence of COVID-19 in China [78].

Apart from the utilization of AI models in predicting studies, they have been also reported as powerful tools to diagnose the infected cases by processing X-ray imaging of the chest to avoid human errors [79,80]. The most common reported AI approaches to detect COVID-19 infections based on X-ray or CT images are CNN models [81,82,83,84]. Aswathy et al. [85] used two CNN models, namely ResNet-50 and DenseNet-201, to identify and assess the COVID-19 infection from CT images as well as the severity condition of the patient. They succeeded in developing a single architecture of the model that can be used to achieve both targets. Li et al. [86] utilized three other CNN models, namely VGG-16, LeNet-5 and ResNet-18, to detect COVID-19 infection using X-ray images. EfficientNet CNN was used to classify the diagnosed cases into normal, pneumonia andCOVID-19 cases based on their X-ray images [87]. CoroNet CNN was used to classify the diagnosed cases into normal, pneumonia-bacterial, pneumonia-viral and COVID--19 cases based on their X-ray images [88]. ResNet50 and VGG16 CCN were utilized to detect COVID-19 infections based on CT images [89]. The proposed models exhibited excellent accuracy when they were used as binary classifiers (normal and COVID-19), but their accuracy degraded when they were used as multiclass classifiers (normal, pneumonia, COVID-19). The same models (ResNet-50, VGG-16) along with auto-encoder CNN and machine learning techniques, such as nearest neighbor, logical regression, support vector machine, random forest and stochastic gradient descent, were proposed to classify CT images of COVID-19 [90]. A hybrid CNN, Sobel filter and support vector machine model was developed to detect COVID-19 based on X-ray images [91]. First, X-ray images are filtered using Sobel filter to detect the edges and contours of the images. Then, the filtered images are fed into to CNN model and classified using a support vector machine. Saha et al. [92] proposed a hybrid AIT to detect COVID-19 infection from X-ray images. First, a CNN model was utilized to extract the main features of X-ray images. Then, machine learning approaches such as support vector machine, random forest, and decision tree were utilized to detect COVID-19 infection based on the extracted features. Another hybrid CNN and deep neural network was developed to examine CT images to detect COVID-19 infections [93]. Shorfuzzaman and Hossain [94] developed a Siamese neural network model to detect COVID-19 infections using X-ray images. A fine-tuned VGG16 CNN was used as an encoder to detect unbiased feature in examined images. The diagnosis of the COVID-19 from the images was formulated as N-way/K-shot classification problem where N and K denote the number of data samples and class labels used to train the model. The model succeeded in obtaining reasonable accuracy using a limited number of samples and examples during the training process. The integration between AI models and the metaheuristic optimizers [95,96] has been also reported as an efficient tool to diagnose COVID-19 infections.

More investigations should be carried out to develop hybrid AI models to predict and forecast the prevalence of this pandemic as well as detect the infected cases via image processing approaches. These hybrid models are composed of an AI model, such as ANN, ANFIS and RVFL integrated with metaheuristic optimizers [96] such as pigeon optimizer [97], gradient-based optimizer [98], parasitism-predation optimizer [99], ecosystem-based optimization [100], Hunger games algorithm [101], flower pollination algorithm [102] and political optimizer [103].

## 5. Conclusions

This study presents a review on the applications of AI techniques for forecasting the prevalence of the COVID-19 pandemic. The basics and the mathematical formulation of different AI approaches used in this context are presented, including nonlinear autoregressive neural network, adaptive neuro-fuzzy inference system, hybrid fractal-fuzzy approach, long short-term memory network, Bayesian neural network, variational auto-encoder and singular spectrum analysis. Different statistical measures used to evaluate the forecasting accuracy of the AI approaches are discussed. Despite of the differences in human behavior, applied measures and available data for each country, AI approaches had much higher forecasting accuracy compared to conventional statistical approaches. The evidence from the current review indicates that there is not a single approach that may be considered as the best one for all investigated countries, which have different pandemic trends. Moreover, the integration between AI forecasting approaches and advanced optimization methods could significantly enhance the accuracy of the whole model with acceptable increase in the computational cost. Thus, it is recommended to use hybrid models instead of standalone models. A substantial concern should be paid towards the utilization of AI approaches to capture the sudden variation in the Covid-19 spread caused by the applied motion control strategies. Future work may focus on the integration between AI approaches and metaheuristic optimization methods to enhance their forecasting accuracy. Forecasting the trend of the economic recovery after this pandemic deserves the researchers’ attention.
